# Necrological

**Published:** 1881-08

**Authors:** 


### NECROLOGICAL.

"Death is the crown of life:
Were death denied, poor man would live in vain."

Dr. Greensville Duvell, for many years Professor of Surgery in the Texas Medical College, died in Galveston June the 9th, 1881, after an illness of only five days. Dr. Duvell was a native of Virginia, having been born in that State in 1826. He originated a method for the treatment of hernia that bears his name, and was the author of a work upon surgery, and one on yellow fever. We received a letter from him some weeks ago in advocacy of the new system of spelling, and, incidentally, of the metric system of weights and measures for use in this country. He was a surgeon in the Confederate army, and for many years editor of a medical journal.

Dr. Hugh Lenox Hodge, son of the late distinguished Prof. Hugh L. Hodge, of the University of Pennsylvania, and a highly respectable physician of Philadelphia, died of pneumonia, at his residence in that city, June 9th, 1881. He was born July 30th, 1836. He was a thoroughly educated and accomplished member of the profession, and died deeply regretted by all who knew him. He held the posirion of demonstrator of anatomy in the university, in which his illustrious father was so long an ornament, at the time of his death, and had done so for many years prior to that sad event, to the entire satisfaction of all parties interested in that grand old alma mater. Dr. Hodge contributed many valuable papers to the medical periodicals—some of them are highly esteemed at the present time. He held many positions of responsibility, all of which he discharged with fidelity to all concerned. Truly hath a good man fallen.

The cable, on June 14th, announced the death of Dr. Joseph Skoda, the distinguished professor of Vienna. He was born in Bohemia, in 1805, graduated at the Vienna Medical Schools in 1831, in 1834 became second physician to the general hospital at Vienna, and became successively Physician for the Division of Lung Diseases, Chief Physician of the hospital, Professor of Clinics, and a member of the Vienna Academy of Sciences.

We are pained to learn of the early death of Professor George S. Blackie, M. D. (Edin.) Ph. D., of Nashville, Tenn. The sad event occurred at his home in Nashville, on Sunday morning, 29th of June, 1881. Dr. Blackie was born at Aberdeen, Scotland, April 10th, 1834. He was a man of high order of intellect, and one of the most thoroughly cultured men in the medical profession. He established himself in Nashville in 1857, and the following year was united in marriage with Miss Martha E. Cheatham, a sister of Gen. Frank Cheatham, of Confederate army fame. He was a member of many learned societies, at home and abroad, and a member of most of the high orders of Masonry in the world. He was distinguished as a professor and writer, and to crown the whole a sincere and devoted Christian. Peace to his ashes.

We are pained to record the demise of Dr. John Waugh, of Baltimore. The sad event occurred at his late residence on Carrolton Avenue, on July 6th, after an illness of eight days duration. Dr. Waugh was enjoying his usual good health up, almost, to the very hour when he was stricken with pneumonia of the left lung. The attack was arrested with unusual promptness ; however, when exposed to a draft of air, during the then existing very hot weather, developed the fatal disease in the right lung, which steadily ran its course to a fatal termination, notwithstanding the best skill and attention of his medical attendants. Dr. Waugh was in the 53d year of his age, and the youngest son of the late distinguished Bishop Beverly Waugh, D. D., of the M. E. Church. With such a father, and the training he received in his early life, it is not surprising to know that he reached maturity a most earnest and exemplary Christian gentleman, and that he "Died the death of the righteous." He received his medical education at the University of Maryland, and was greatly esteemed for his medical skill and exalted Christian character.

